# Research Landscape of Acquired Dermal Macular Hyperpigmentation: A Bibliometric Analysis

**DOI:** 10.1155/drp/8871423

**Published:** 2025-07-15

**Authors:** Abdulaziz Hamid, Kara Turner, Nada Elbuluk

**Affiliations:** ^1^Medical College of Wisconsin, Milwaukee, Wisconsin, USA; ^2^Albert Einstein College of Medicine, Bronx, New York, USA; ^3^Department of Dermatology, University of Southern California Keck School of Medicine, Los Angeles, California, USA

**Keywords:** acquired dermal macular hyperpigmentation, ashy dermatosis, bibliometric analysis, erythema dyschromicum perstans, lichen planus pigmentosus, Riehl's melanosis

## Abstract

**Background:** Acquired dermal macular hyperpigmentation (ADMH) includes lichen planus pigmentosus (LPP), ashy dermatosis (erythema dyschromicum perstans), and Riehl's melanosis (pigmented contact dermatitis/pigmented cosmetic dermatitis). The conditions that make up ADMH overlap in clinical and histopathological features.

**Objective:** To conduct a bibliometric analysis to identify the top 100 most cited publications in ADMH.

**Methods:** A Web of Science search was conducted on September 18, 2024, using the search terms “lichen planus pigmentosus,” “ashy dermatosis,” “erythema dyschromicum perstans,” “riehl melanosis,” “pigmented cosmetic dermatitis,” “pigmented contact dermatitis,” “acquired dermal macular hyperpigmentation,” or “acquired macular pigmentation of unknown aetiology” in the title or abstract of articles published between 1998 and 2024. The search was filtered to include articles, letters, reviews, and editorials in English. Data collected included title, author, publication year, times cited, journal of publication, affiliations, and country of origin. The top 100 most cited publications were ranked based on annual citation score.

**Results:** The top 100 most cited publications consisted of 62 articles, 24 letters (i.e., letter to the editor and comments), 8 editorials, and 6 reviews published between 1998 and 2023. The most articles were published in 2018 with 14 publications. The top contributing journals were the *International Journal of Dermatology* (*n* = 15, 15%) and the *Journal of the European Academy of Dermatology and Venereology* (*n* = 14, 14%). India, South Korea, and the United States contributed the most publications (*n* = 61, 61%) on ADMH (32, 17, and 12, respectively). India also led in having the top three corresponding authors, Muthu Sendhil Kumaran (*n* = 8, 8%), Keshavamurthy Vinay (*n* = 4, 4%), and Vinod Kumar Sharma (*n* = 3, 3%).

**Conclusion:** This bibliometric analysis reveals a geographical concentration in ADMH research, emphasizing the need for increased research on these conditions with more global representation in future studies.

## 1. Introduction

Acquired dermal macular hyperpigmentation (ADMH) includes pigmentary disorders such as lichen planus pigmentosus (LPP), ashy dermatosis (erythema dyschromicum perstans), and Riehl's melanosis (pigmented contact dermatitis/pigmented cosmetic dermatitis) [1]. The term “acquired dermal macular hyperpigmentation” was introduced in 2017 to provide a common umbrella term for these conditions [2]. These conditions are grouped together due to shared clinical and histopathological features including color of pigmentation, macule and patch morphology, and histopathology features of pigment incontinence and interface dermatitis [3]. In a Delphi consensus, over 80% of expert researchers in the field of pigmentary diseases agreed on the umbrella term “acquired dermal macular hyperpigmentation” to classify these conditions [1]. ADMH commonly affects individuals with darker skin and can impact the quality of life for those affected [4, 5]. Jiang et al. [6] conducted a systematic review of quality of life in patients with ADMH (LPP, ashy dermatosis, and Riehl's melanosis) looking at seven studies with 259 patients all of which reported impaired quality of life. Five of the seven studies used the Dermatology Life Quality Index and found that ADMH generally has a “moderate effect on patient's life” [6]. Four studies suggest that the impact of ADMH on quality of life may be greater than that of melasma, while two studies indicate that its impact is likely similar to that of vitiligo [6]. To our knowledge, this is the first bibliometric analysis in ADMH, a group of challenging pigmentary conditions with limited research. Bibliometric analysis is a systematic approach to examining scientific literature by identifying patterns, trends, and impacts within a specific field [7]. This analysis allows researchers to identify the most cited publications in a field as well as highlight areas that can be explored in future studies [7].

## 2. Materials and Methods

A search was conducted using the Web of Science (WoS) Core Collection on September 18, 2024, with the following search terms: “lichen planus pigmentosus” or “ashy dermatosis” or “erythema dyschromicum perstans” or “riehl melanosis” or “pigmented cosmetic dermatitis” or “pigmented contact dermatitis” or “acquired dermal macular hyperpigmentation” or “acquired macular pigmentation of unknown aetiology” in the title or abstract of the articles. There were no limitations on the publication year. This search resulted in 649 articles. The search was refined by filtering for document types in English including article, letter (i.e., letter to the editor and comments), review article, editorial material, and early access resulting in a total of 581 articles. The results were sorted from highest to lowest citation count (Times Cited, WoS Core). Articles were included in the bibliometric analysis if the central focus was on one or more of the ADMH conditions. Variants of LPP, such as LPP inversus, were also included. Articles discussing ADMH conditions alongside other pigmentary disorders (e.g., melasma and vitiligo) were included if ADMH was part of the main focus of the study. However, articles with limited discussion of ADMH were excluded.

The top 100 most cited publications were first selected based on the highest citation count. The annual citation scores for these top 100 articles were calculated by dividing the citation count by the number of years since publication. The 100 most cited publications were then ranked by annual citation count. The annual citation scores were calculated to account for differences in time of publication. Publications with the same annual citation count were ranked by publication date with more recent articles ranked higher. When two articles had the same annual citation count and publication year, the article published in the higher impact factor journal was ranked higher.

The top 100 most cited publications were directly analyzed in the WoS and exported to Excel for further analysis. For each article, data were collected on the title, author, publication year, times cited, journal of publication, affiliations, and country of origin.

## 3. Results

A total of 581 articles were obtained from the WoS Core Collection for review. The top 100 most cited publications included 62 articles, 24 letters, 8 editorials, and 6 reviews (Supporting Table 1) [1, 3–5, 8–40], [41–80], [81–103].

### 3.1. Publication Years

**Figure 1:**
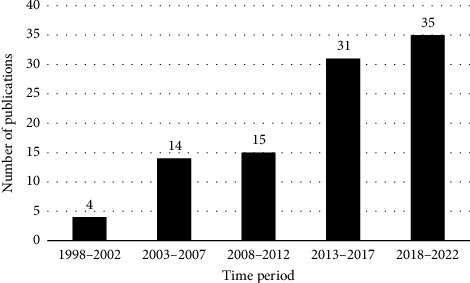
Trends in ADMH publications. Figure legend: Trends are displayed in 5 year periods. Note there was one publication from 2023 not shown in the figure.

The top 100 most cited publications were published between 1998 and 2023. The year in which the most articles were published was 2018 with 14 articles. The oldest of these publications “Erythema dyschromicum perstans: response to dapsone therapy” by Kontochristopoulos et al. was published in October 1998 in the *International Journal of Dermatology*. The most recent of these articles is “A Delphi consensus on the nomenclature and diagnosis of lichen planus pigmentosus and related entities” by Sarkar et al. published in January 2023 in the *Indian Journal of Dermatology Venereology & Leprology.* The number of publications gradually increased across each time period (Figure 1). There were 4 publications between 1998 and 2002, followed by 14 publications between 2003 and 2007 and 15 publications between 2008 and 2012. There were 31 publications between 2013 and 2017 and a peak of 35 publications between 2018 and 2022.

### 3.2. Citations

The top 100 most cited publications totaled 1991 citations, ranging from 7 to 99. The most cited publication with a total of 99 citations was a retrospective study on the clinical and epidemiological features and histopathological findings of 124 patients with LPP by Kanwar et al. published in the *Clinical and Experimental Dermatology* in 2003 titled “A study of 124 Indian patients with lichen planus pigmentosus.” The most cited publication based on the annual citation score (12.5) was a review article on LPP, and its variants by Robles-Méndez et al. published in the *International Journal of Dermatology* in 2018 titled “Lichen planus pigmentosus and its variants: review and update.”

### 3.3. Journals, Countries/Regions, Authors, and Institutions

The top 100 most cited publications were from 34 different journals. The top three journals were the *International Journal of Dermatology* (*n* = 15, 15%), *the Journal of the European Academy of Dermatology and Venereology* (*n* = 14, 14%), and *Indian Journal of Dermatology, Venereology, and Leprology* (*n* = 9, 9%) (Table 1). Together these three journals included 38% of all ADMH publications. The countries contributing to the most publications on ADMH were India (*n* = 32, 32%), South Korea (*n* = 17, 17%), and the United States (*n* = 12, 12%), collectively making up 61% of the total contributions across all publications. When focusing specifically on the corresponding author's country of origin, the top four countries were India (*n* = 31, 31%), South Korea (*n* = 16, 16%), the United States (*n* = 7, 7%), and China (*n* = 7, 7%) (Table 2). India also led in having the top three corresponding authors, Muthu Sendhil Kumaran (*n* = 8, 8%), Keshavamurthy Vinay (*n* = 4, 4%), and Vinod Kumar Sharma (*n* = 3, 3%). The top three affiliations with the most publications were Postgraduate Institute of Medical Education Research (PGIMER), Chandigarh in India (*n* = 17, 17%), All India Institute of Medical Sciences (AIIMS), New Delhi, in India (*n* = 5, 5%), and Seoul National University (SNU) in South Korea (*n* = 5, 5%).

### 3.4. Keywords

**Figure 2:**
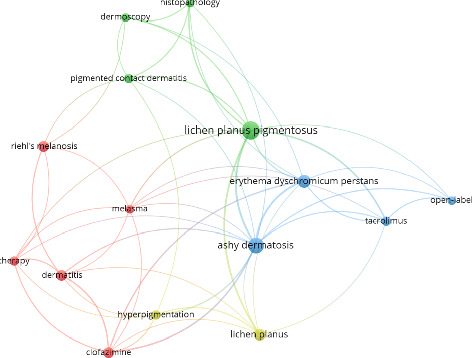
Keyword network in ADMH literature. Figure legend: Keyword networks are visual representations of keyword relationships. Each node represents a keyword, with node size proportional to its frequency of occurrence. Lines that connect the nodes demonstrate relationships between keywords, and thicker lines indicate keywords that appear more frequently together. Colors represent different clusters. The keyword network is made up of four clusters. The green cluster includes keywords “dermoscopy,” “histopathology,” “lichen planus pigmentosus,” and “pigmented contact dermatitis.” This cluster focuses on diagnostic techniques for ADMH conditions. The blue cluster includes “ashy dermatosis,” “erythema dyschromicum perstans,” “tacrolimus,” and “open label.” This cluster focuses on treatment for ADMH conditions, highlighting open-label studies. The red cluster includes “melasma,” “Riehl's melanosis,” “dermatitis,” “clofazimine,” and “therapy.” This cluster also focuses on treatment for ADMH conditions. The yellow cluster includes “lichen planus” and “hyperpigmentation.” This cluster represents a broader view of ADMH.

In total, 231 keywords were associated with the articles analyzed, of which 15 occurred 5 or more times in ADMH literature. VOSviewer software (Leiden, Netherlands) was utilized to generate a keyword co-occurrence network (Figure 2). The top keywords include “lichen planus pigmentosus” (*n* = 21), “ashy dermatosis” (*n* = 17), and “erythema dyschromicum perstans” (*n* = 11). In addition, the keywords “lichen planus” (*n* = 9), “riehl's melanosis” (*n* = 8), “dermatitis” (*n* = 8), and “clofazimine” (*n* = 8) were also frequently used. Other keywords “hyperpigmentation,” “therapy,” and “tacrolimus” each occurred 6 times, while “histopathology,” “melasma,” and “pigmented contact dermatitis” occurred 5 times. This keyword analysis provides insight into the major themes that drive research in ADMH.

### 3.5. Research Focus Areas

Each of the top 100 most cited publications was assigned to a single primary category based on the article's main focus. The most common categories were clinical and treatment (*n* = 22 each), reflecting a strong focus on disease presentation and treatment strategies (Table 3). Etiology was the next most frequent category (*n* = 16), followed by investigations (*n* = 15), which included methods such as dermoscopy and immunohistochemistry, and then semantics (*n* = 14). Few studies focused on epidemiology (*n* = 5), histopathology (*n* = 4), and quality of life (*n* = 2), highlighting potential research areas within ADMH.

## 4. Discussion

This bibliometric analysis identified key findings in the literature on ADMH. Over the past 2 decades, there has been an increase in publications on ADMH. India contributed the most publications, followed by South Korea and the United States. Notably, the top three corresponding authors were all from India. The large number of Indian researchers and India's leading contribution to ADMH publications supports the finding that these conditions may occur at higher rates in populations of color and certain parts of the world including India. It also suggests that this condition may be viewed as bearing greater research importance and quality of life effects in India given the increased number of publications from there.

Pigmentary disorders have been found to have a negative impact on the quality of life of the affected individuals [5]. In this bibliometric analysis of ADMH, only two articles from the top 100 most cited publications focused on quality of life or the psychological impact of these conditions on patients. Interestingly, one of these articles which is ranked second among the top 100 most cited publications in ADMH is entitled “Psychological disturbances in patients with pigmentary disorders: a cross-sectional study” by Dabas et al. published in February 2020 in the J*ournal of the European Academy of Dermatology and Venereology*. Dabas et al. [5] found an increased prevalence of anxiety, depression, and somatoform disorders in patients with ADMH. The second study by Yadav A et al. was published in July 2018 in the *Journal of Cosmetic Dermatology* and is ranked 24th from the top 100 most cited publications in ADMH. Entitled “Quality of life in patients with acquired pigmentation: An observational study,” the study found that patients with LPP and pigmented contact dermatitis experienced moderate to very large impacts on their quality of life [28]. These two studies highlight the significant negative impact of ADMH on patients' psychological well-being and quality of life. It is noteworthy that, for both articles, the corresponding author is from India. Given the scarcity of literature on the quality of life and psychological aspects of ADMH, it would be beneficial to have further increased research on these areas from different countries around the world.

## 5. Limitations

Our bibliometric analysis was limited to using only the WoS Core Collection database and English-language publications. As a result, some non-English regional publications, particularly from Asian countries where ADMH is more prevalent, may have been excluded. However, as one of the most widely used and comprehensive global databases the WoS Core Collection is likely to capture the most influential and scientifically rigorous research on ADMH. Furthermore, focusing on the top 100 most cited publications may have excluded more recent studies with lower citation counts since they have not yet accumulated sufficient citations.

## 6. Conclusion

This bibliometric analysis reveals a geographical concentration in ADMH research, with most publications arising from a few countries, majority of which are in Asia. This highlights the need for increased research on ADMH with more global representation. Expanding research efforts to other regions could improve our international understanding of these conditions. Additionally, future studies should include consideration of quality of life and the psychological effects of ADMH.

## Figures and Tables

**Table 1:** Top 5 journals for the 100 most cited publications in ADMH.

Journal	Frequency	Impact factor^∗^
International Journal of Dermatology	15	3.5
Journal of the European Academy of Dermatology and Venereology	14	8.4
Indian Journal of Dermatology Venereology & Leprology	9	3.2
Journal of the American Academy of Dermatology	5	12.8
Journal of Dermatology	5	2.9

^∗^The journal impact factor was collected from the Web of Science database.

**Table 2:** Corresponding author's country of origin for the top 100 most cited publications in ADMH.

Countries	Frequency^∗^
India	31
South Korea	16
United States, China	7
Japan, Spain	5
Turkey, Brazil, Singapore	3
Mexico, Australia, Netherlands, Tunisia, Thailand	2
Colombia, Greece, Iran, Czech Republic, Lebanon, Portugal, Italy, Kuwait, South Africa, Israel	1

^∗^All countries listed in the same row have the same frequency.

**Table 3:** Primary topics covered in the 100 most cited publications in ADMH literature.

Primary topic	Number of publications
Clinical	22
Treatment	22
Etiology	16
Investigations	15
Semantics	14
Epidemiology	5
Histopathology	4
Quality of life	2

## Data Availability

The data that support the findings of this study are available from the corresponding author upon reasonable request.

## Supporting Information

Supporting Table 1. Top 100 most cited publications in ADMH literature from 1998 to 2023. Footnote: ^∗^The journal impact factor was collected from the Web of Science database.
