# Supplementary material for: Research Landscape of Acquired Dermal Macular Hyperpigmentation: A Bibliometric Analysis
Source: Dermatol Res Pract. 2025 Jul 15;2025:8871423. doi: 10.1155/drp/8871423 (PMC12283195; doi:10.1155/drp/8871423)
Supplement: Supporting Information — Additional supporting information can be found online in the Supporting Information section. [file 8871423.f1.docx]

**Description:** Top 100 most cited publications in ADMH literature from 1998 to 2023

**Supplementary Table 1**. Top 100 most cited publications in ADMH literature from 1998 to 2023

| **Rank** | **Article Title** | **Times Cited** | **Publication Year** | **Annual Citation Count** | **First Author** | **Journal** | **Impact factor*** | **Country** |
| --- | --- | --- | --- | --- | --- | --- | --- | --- |
| 1 | Lichen planus pigmentosus and its variants: review and update | 75 | 2018 | 12.5 | Robles-Méndez, JC^8^ | International Journal of Dermatology | 3.5 | Mexico |
| 2 | Psychological disturbances in patients with pigmentary disorders: a cross-sectional study | 43 | 2020 | 10.8 | Dabas, G^5^ | Journal of the European Academy of Dermatology and Venereology | 8.4 | India |
| 3 | A Delphi consensus on the nomenclature and diagnosis of lichen planus pigmentosus and related entities | 10 | 2023 | 10 | Sarkar, R^1^ | Indian Journal of Dermatology Venereology & Leprology | 3.2 | India |
| 4 | A global consensus statement on ashy dermatosis, erythema dyschromicum perstans, lichen planus pigmentosus, idiopathic eruptive macular pigmentation, and Riehl's melanosis | 48 | 2019 | 9.6 | Kumarasinghe, SPW^9^ | International Journal of Dermatology | 3.5 | Australia |
| 5 | Clinical and dermoscopic features of lichen planus pigmentosus in 37 patients with frontal fibrosing alopecia | 50 | 2016 | 6.3 | Pirmez, R^10^ | British Journal of Dermatology | 11 | Brazil |
| 6 | Dermatoscopic evaluation and histopathological correlation of acquired dermal macular hyperpigmentation | 42 | 2017 | 6 | Vinay, K^11^ | International Journal of Dermatology | 3.5 | India |
| 7 | Frontal fibrosing alopecia and lichen planus pigmentosus: is there a link? | 66 | 2013 | 6 | Dlova, NC^12^ | British Journal of Dermatology | 11 | South Africa |
| 8 | Pigmented contact dermatitis due to kojic acid. A paradoxical side effect of a skin lightener | 75 | 2010 | 5.4 | García-Gavín, J^13^ | Contact Dermatitis | 4.8 | Spain |
| 9 | Low-dose oral isotretinoin therapy in lichen planus pigmentosus: an open-label non-randomized prospective pilot study | 42 | 2016 | 5.3 | Muthu, SK^14^ | International Journal of Dermatology | 3.5 | India |
| 10 | Clinicopathological characteristics of lichen planus pigmentosus and its response to tacrolimus ointment: an open label, non-randomized, prospective study | 72 | 2010 | 5.1 | Al-Mutairi, N^15^ | Journal Of the European Academy of Dermatology and Venereology | 8.4 | Kuwait |
| 11 | Optimizing Q-switched lasers for melasma and acquired dermal melanoses | 24 | 2019 | 4.8 | Aurangabadkar, SJ^16^ | Indian Journal of Dermatology Venereology & Leprology | 3.2 | India |
| 12 | A study of 124 Indian patients with lichen planus pigmentosus | 99 | 2003 | 4.7 | Kanwar, AJ^17^ | Clinical and Experimental Dermatology | 3.7 | India |
| 13 | A pilot study for triple combination therapy with a low-fluence 1064nm Q-switched Nd:YAG laser, hydroquinone cream and oral tranexamic acid for recalcitrant Riehl's Melanosis | 31 | 2017 | 4.4 | Kwon, HH^18^ | Journal of Dermatological Treatment | 2.9 | South Korea |
| 14 | Therapeutic Effects of New Pulsed-Type Microneedling Radiofrequency for Refractory Facial Pigmentary Disorders | 8 | 2022 | 4 | Park, BJ^19^ | Dermatologic Surgery | 2.5 | South Korea |
| 15 | Contact sensitization to hair colours in acquired dermal macular hyperpigmentation: results from a patch and photo-patch test study of 108 patients | 20 | 2019 | 4 | Bishnoi, A^20^ | Journal Of the European Academy of Dermatology and Venereology | 8.4 | India |
| 16 | Dermoscopy and patch testing in patients with lichen planus pigmentosus on face: A cross-sectional observational study in fifty Indian patients | 28 | 2017 | 4 | Sharma, VK^21^ | Indian Journal of Dermatology Venereology & Leprology | 3.2 | India |
| 17 | Ribociclib-Induced Erythema Dyschromicum Perstans (Ashy Dermatosis)-Like Pigmentation in a Metastatic Breast Cancer Patient | 11 | 2021 | 3.7 | Mariano, M^22^ | Journal of Breast Cancer | 2.2 | Italy |
| 18 | A pilot study of oral tranexamic acid and Glycyrrhizin compound in the treatment of recalcitrant Riehl's melanosis | 18 | 2019 | 3.6 | Xu, ZY^23^ | Journal of Cosmetic Dermatology | 2.3 | China |
| 19 | Case of lichen planus pigmentosus-inversus after Oxford-AstraZeneca COVID-19 vaccine: cause or coincidence? | 7 | 2022 | 3.5 | Sun, L^24^ | Journal of the European Academy of Dermatology and Venereology | 8.4 | Portugal |
| 20 | Non-Ablative 1927 nm Fractional Thulium Fiber Laser: New, Promising Treatment Modality for Riehl's Melanosis | 10 | 2021 | 3.3 | Kim, SM^25^ | Lasers in Surgery and Medicine | 2.2 | South Korea |
| 21 | Acquired Dermal Macular Hyperpigmentation: An Update | 10 | 2021 | 3.3 | Vinay, K^3^ | Indian Dermatology Online Journal | 1.9 | India |
| 22 | Four views of Riehl's melanosis: clinical appearance, dermoscopy, confocal microscopy and histopathology | 32 | 2014 | 3.2 | Wang, L^26^ | Journal of the European Academy of Dermatology and Venereology | 8.4 | China |
| 23 | Macular pigmentation of uncertain aetiology revisited: two case reports and a proposed algorithm for clinical classification | 22 | 2017 | 3.1 | Chandran, V^27^ | Australasian Journal of Dermatology | 2.2 | Australia |
| 24 | Quality of life in patients with acquired pigmentation: An observational study | 18 | 2018 | 3 | Yadav, A^28^ | Journal of Cosmetic Dermatology | 2.3 | India |
| 25 | Lichen Planus Pigmentosus: The Controversial Consensus | 23 | 2016 | 2.9 | Ghosh, A^29^ | Indian Journal of Dermatology | 1 | India |
| 26 | Frontal fibrosing alopecia and lichen planus pigmentosus | 29 | 2014 | 2.9 | Berliner, JG^30^ | Journal of the American Academy of Dermatology | 12.8 | United States |
| 27 | A clinico-demographic study of 344 patients with lichen planus pigmentosus seen in a tertiary care center in India over an 8-year period | 11 | 2020 | 2.8 | Vinay, K^31^ | International Journal of Dermatology | 3.5 | India |
| 28 | Lichen planus pigmentosus-inversus | 64 | 2001 | 2.8 | Pock, L^32^ | Journal of the European Academy of Dermatology and Venereology | 8.4 | Czech Republic |
| 29 | Combination therapy with salicylic acid chemical peels, glycyrrhizin compound, and vitamin C for Riehl's melanosis | 10 | 2020 | 2.5 | Wang, L^33^ | Journal of Cosmetic Dermatology | 2.3 | China |
| 30 | Lichen planus pigmentosus: a retrospective clinico-epidemiologic study with emphasis on the rare follicular variant | 20 | 2016 | 2.5 | Sindhura, KBN^34^ | Journal Of the European Academy of Dermatology and Venereology | 8.4 | India |
| 31 | Lichen planus pigmentosus inversus: a series of 10 Tunisian patients | 19 | 2016 | 2.4 | Mohamed, M^35^ | International Journal of Dermatology | 3.5 | Tunisia |
| 32 | Clinical, Histopathological Characteristics and Immunohistochemical Findings in Lichen Planus Pigmentosus | 16 | 2017 | 2.3 | Bhat, RM^36^ | Indian Journal of Dermatology | 1 | India |
| 33 | Two Japanese cases of lichen planus pigmentosus-inversus | 38 | 2007 | 2.2 | Kashima, A^37^ | International Journal of Dermatology | 3.5 | Japan |
| 34 | Coexistence of frontal fibrosing alopecia with lichen planus pigmentosus | 21 | 2014 | 2.1 | Rao, R^38^ | International Journal of Dermatology | 3.5 | India |
| 35 | Patch testing and Histopathology in Thai patients with hyperpigmentation due to Erythema dyschromicum perstans, Lichen planus pigmentosus, and Pigmented contact dermatits | 21 | 2014 | 2.1 | Tienthavorn, T^39^ | Asian Pacific Journal of Allergy and Immunology | 2.3 | Thailand |
| 36 | Successful Treatment of Riehl's Melanosis With Mid-Fluence Q-Switched Nd:YAG 1064-nm Laser | 8 | 2020 | 2 | Cho, MY^40^ | Lasers in Surgery and Medicine | 2.2 | South Korea |
| 37 | Lichen Planus Pigmentosus: A Clinico-etiological Study | 10 | 2019 | 2 | Mendiratta, V^41^ | Indian Dermatology Online Journal | 1.9 | India |
| 38 | Clinical, dermoscopic, and trichoscopic analysis of frontal fibrosing alopecia associated with acquired dermal macular hyperpigmentation: A cross sectional observational case-control study | 12 | 2018 | 2 | Kumaran, MS^42^ | Journal Of the American Academy of Dermatology | 12.8 | India. |
| 39 | Low-pulse energy Q-switched Nd:YAG laser treatment for hair-dye-induced Riehl's melanosis | 17 | 2015 | 1.9 | On, HR^43^ | Journal of Cosmetic and Laser Therapy | 1.2 | South Korea |
| 40 | Ashy dermatoses - a critical review of the literature and a proposed simplified clinical classification | 30 | 2008 | 1.9 | Zaynoun, S^44^ | International Journal of Dermatology | 3.5 | Lebanon |
| 41 | Ashy Dermatosis and Lichen Planus Pigmentosus: The Histopathological Differences | 9 | 2019 | 1.8 | Rutnin, S^45^ | Biomed Research International | 2.6 | Thailand |
| 42 | Significant reduction in the expression of interleukins-17A, 22 and 23A, forkhead box p3 and interferon gamma delineates lichen planus pigmentosus from lichen planus | 9 | 2019 | 1.8 | Kumaran, MS^46^ | Archives of Dermatological Research | 1.8 | India |
| 43 | An open-label non-randomized prospective pilot study of the efficacy of Q-switched Nd-YAG laser in management of facial lichen planus pigmentosus | 9 | 2019 | 1.8 | Shah, SD^47^ | Journal of Cosmetic and Laser Therapy | 1.2 | India |
| 44 | A novel scale for measurement of acquired dermal macular hyperpigmentation severity | 11 | 2018 | 1.8 | Vinay, K^48^ | Journal of the European Academy of Dermatology and Venereology | 8.4 | India |
| 45 | Ashy dermatosis, lichen planus pigmentosus and pigmented cosmetic dermatitis: Are we splitting the hair? | 11 | 2018 | 1.8 | Gupta, V^4^ | Indian Journal of Dermatology Venereology & Leprology | 3.2 | India |
| 46 | Acquired diffuse slate-grey facial dyspigmentation due to henna: an unrecognized cause of pigment contact dermatitis in Korean patients | 11 | 2018 | 1.8 | Woo, YR^49^ | European Journal of Dermatology | 2 | South Korea |
| 47 | Lichen planus pigmentosus inversus | 20 | 2013 | 1.8 | Barros, HR^50^ | Anais Brasileiros De Dermatologia | 2.6 | Brazil |
| 48 | A Pilot Study of Intense Pulsed Light in the Treatment of Riehl's Melanosis | 23 | 2011 | 1.8 | Li, YH^51^ | Dermatologic Surgery | 2.5 | China |
| 49 | Erythema dyschromicum perstans in children: a report of 14 cases | 35 | 2005 | 1.8 | Torrelo, A^52^ | Journal of the European Academy of Dermatology and Venereology | 8.4 | Spain |
| 50 | Lichen planus pigmentosus-inversus: Report of three Chinese cases and review of the published work | 15 | 2015 | 1.7 | Chen, SM^53^ | Journal of Dermatology | 2.9 | China |
| 51 | A Case of Linear Lichen Planus Pigmentosus | 24 | 2010 | 1.7 | Seo, JK^54^ | Annals of Dermatology | 1.5 | South Korea |
| 52 | Reliability assessment and validation of the dermal pigmentation area and severity index: a new scoring method for acquired dermal macular hyperpigmentation | 8 | 2019 | 1.6 | Kumaran, MS^55^ | Journal of the European Academy of Dermatology and Venereology | 8.4 | India |
| 53 | Everything is in the name: Macular hyperpigmentation of uncertain etiology or acquired dermal macular hyperpigmentation of varied etiologies? | 8 | 2019 | 1.6 | Bishnoi, A^56^ | Indian Journal of Dermatology Venereology & Leprology | 3.2 | India |
| 54 | Axillary lichen planus pigmentosus-inversus: Dermoscopic clues of a rare entity | 16 | 2014 | 1.6 | Murzaku, EC^57^ | Journal of the American Academy of Dermatology | 12.8 | United States |
| 55 | Lichen planus pigmentosus-inversus occurring extensively in multiple intertriginous areas | 19 | 2012 | 1.6 | Ohshima, N^58^ | Journal of Dermatology | 2.9 | Japan |
| 56 | Clinical Profile and Allergens in Pigmented Cosmetic Dermatitis and Allergic Contact Dermatitis to Cosmetics in India | 9 | 2018 | 1.5 | Sharma, VK^59^ | Dermatitis | 4 | India |
| 57 | A retrospective clinico-pathological study comparing lichen planus pigmentosus with ashy dermatosis | 9 | 2018 | 1.5 | Cheng, HM^60^ | Australasian Journal of Dermatology | 2.2 | Singapore |
| 58 | A pilot study of a novel dual - pulsed 1064 nm Q-switched Nd: YAG laser to treat Riehl's melanosis | 15 | 2014 | 1.5 | Chung, BY^61^ | Journal of Cosmetic and Laser Therapy | 1.2 | South Korea |
| 59 | Linear lichen planus pigmentosus of the forehead treated by neodymium:yttrium-aluminum-garnet laser and topical tacrolimus | 18 | 2012 | 1.5 | Kim, JE^62^ | Journal of Dermatology | 2.9 | South Korea |
| 60 | A retrospective study of lichen planus pigmentosus with focus on palmoplantar involvement | 7 | 2019 | 1.4 | Dabas, G^63^ | Clinical and Experimental Dermatology | 3.7 | India |
| 61 | Analysis of Clinical Features and Treatment Outcomes Using 1,064-nm Nd-YAG Laser with Topical Hydroquinone in Patients with Riehl's Melanosis: A Retrospective Study in 10 Patients | 7 | 2019 | 1.4 | Choi, CW^64^ | Annals of Dermatology | 1.5 | South Korea |
| 62 | Lichen planus pigmentosus inversus | 14 | 2014 | 1.4 | Ghorbel, HH^65^ | Indian Journal of Dermatology Venereology & Leprology | 3.2 | Tunisia |
| 63 | Kumkum-induced dermatitis: an analysis of 46 cases | 23 | 2007 | 1.4 | Nath, AK^66^ | Clinical and Experimental Dermatology | 3.7 | India |
| 64 | Erythema dyschromicum perstans: the continuing enigma of Cinderella or ashy dermatosis | 27 | 2004 | 1.4 | Schwartz, RA^67^ | International Journal of Dermatology | 3.5 | United States |
| 65 | Lichen planus pigmentosus - An appraisal | 8 | 2018 | 1.3 | Kumaran, MS^68^ | International Journal of Dermatology | 3.5 | India |
| 66 | Corneal Involvement by Lichen Planus Pigmentosus | 8 | 2018 | 1.3 | Soleimani, M^69^ | Ocular Immunology and Inflammation | 2.6 | Iran |
| 67 | Frontal Fibrosing Alopecia and Concomitant Lichen Planus Pigmentosus: A Case Series of Seven African American Women | 8 | 2018 | 1.3 | Uwakwe, LN^70^ | Journal of Drugs in Dermatology | 1.5 | United States |
| 68 | Frontal fibrosing alopecia and lichen planus pigmentosus: diagnosis and therapeutic challenge | 9 | 2017 | 1.3 | Mulinari-Brenner, FA^71^ | Anais Brasileiros De Dermatologia | 2.6 | Brazil |
| 69 | Erythema Dyschromicum Perstans Response to Isotretinoin | 10 | 2016 | 1.3 | Wang, F^72^ | JAMA Dermatology | 11.5 | China |
| 70 | Lichen planus pigmentosus-inversus: 5 Turkish cases | 10 | 2016 | 1.3 | Namdar, ND^73^ | Journal of the European Academy of Dermatology and Venereology | 8.4 | Turkey |
| 71 | Clinical and histological aspect of erythema dyschromicum perstans in Korea: A review of 68 cases | 12 | 2015 | 1.3 | Chang, SE^74^ | Journal of Dermatology | 2.9 | South Korea |
| 72 | Two cases of lichen planus pigmentosus inversus: possible causative role of tightly fitting underclothes | 14 | 2013 | 1.3 | Majima, Y^75^ | European Journal of Dermatology | 2 | Japan |
| 73 | Non-ablative 1550 nm fractional laser therapy not effective for erythema dyschromicum perstans and postinflammatory hyperpigmentation: a pilot study | 15 | 2012 | 1.3 | Kroon, MW^76^ | Journal of Dermatological Treatment | 2.9 | Netherlands |
| 74 | Coexistence of classic lichen planus and lichen planus pigmentosus-inversus: resistant to both tacrolimus and clobetasol propionate ointments | 21 | 2008 | 1.3 | Kim, BS^77^ | Journal of the European Academy of Dermatology and Venereology | 8.4 | South Korea |
| 75 | Two cases of lichen planus pigmentosus presenting with a linear pattern | 25 | 2004 | 1.3 | Hong, S^78^ | Journal of Korean Medical Science | 3 | South Korea |
| 76 | Dermoscopy of lichen planus pigmentosus in Indian patients - Pitfalls to avoid | 7 | 2018 | 1.2 | Sonthalia, S^79^ | Indian Journal of Dermatology Venereology & Leprology | 3.2 | India |
| 77 | Detection of lichen planus pigmentosus with dermoscopy and reflectance confocal microscopy | 7 | 2018 | 1.2 | Dai, H^80^ | Skin Research and Technology | 2 | China |
| 78 | Two Cases of Pigmented Contact Dermatitis Caused by Pure Henna Hair Dyes | 7 | 2018 | 1.2 | Shin, JW^81^ | Annals of Dermatology | 1.5 | South Korea |
| 79 | A case of lichen planus pigmentosus that was recalcitrant to topical treatment responding to pigment laser treatment | 11 | 2014 | 1.1 | Han, XD^82^ | Dermatologic Therapy | 3.7 | Singapore |
| 80 | Lichen planus pigmentosus distributed along the lines of Blaschko | 12 | 2013 | 1.1 | Akarsu, S^83^ | International Journal of Dermatology | 3.5 | Turkey |
| 81 | Acquired bilateral melanosis of the neck in perimenopausal women | 13 | 2012 | 1.1 | Park, JY^84^ | British Journal of Dermatology | 11 | South Korea |
| 82 | Erythema dyschromicum perstans: Response to dapsone therapy | 21 | 2004 | 1.1 | Bahadir, S^85^ | International Journal of Dermatology | 3.5 | Turkey |
| 83 | Frontal fibrosing alopecia and extrafacial lichen planus pigmentosum in a caucasian woman | 7 | 2017 | 1 | Franco-Muñoz, M^86^ | Anais Brasileiros De Dermatologia | 2.6 | Spain |
| 84 | Pigmented contact dermatitis | 17 | 2007 | 1 | Shenoi, SD^87^ | Indian Journal of Dermatology Venereology & Leprology | 3.2 | India |
| 85 | Dermoscopic Subpatterns of Ashy Dermatosis Related to Lichen Planus | 12 | 2010 | 0.9 | Vázquez-López, F^88^ | Archives of Dermatology | 4.789 | Spain |
| 86 | Erythema dyschromicum perstans in prepubertal children | 19 | 2003 | 0.9 | Silverberg, NB^89^ | Pediatric Dermatology | 1.2 | United States |
| 87 | Ashy dermatosis-like pigmentation due to ethambutol | 12 | 2008 | 0.8 | Srivastava, N^90^ | Indian Journal of Dermatology Venereology & Leprology | 3.2 | India |
| 88 | Riehl Melanosis Treated Successfully With Q-Switch Nd:YAG Laser | 7 | 2014 | 0.7 | Smucker, JE^91^ | Journal of Drugs in Dermatology | 1.5 | United States |
| 89 | Two Cases of Lichen Planus Pigmentosus-inversus Arising from Long-standing Lichen Planus-inversus | 11 | 2008 | 0.7 | Kim, BS^92^ | Annals of Dermatology | 1.5 | South Korea |
| 90 | Immunopathologic study of erythema dyschromicum perstans (ashy dermatosis) | 12 | 2006 | 0.7 | Vásquez-Ochoa, LA^93^ | International Journal of Dermatology | 3.5 | Colombia |
| 91 | Pigmented Contact Dermatitis Secondary to Benzyl Salicylate | 7 | 2013 | 0.6 | Alagappan, U^94^ | Acta Dermato-Venereologica | 3.5 | Singapore |
| 92 | Formation of Fibrosis After Nonablative and Ablative Fractional Laser Therapy | 7 | 2012 | 0.6 | Wind, BS^95^ | Dermatologic Surgery | 2.5 | Netherlands |
| 93 | Pigmented contact dermatitis due to therapeutic sensitizer as complication of contact immunotherapy in alopecia areata | 9 | 2010 | 0.6 | Inui, S^96^ | Journal of Dermatology | 2.9 | Japan |
| 94 | HLA-DR association with the genetic susceptibility to develop ashy dermatosis in Mexican Mestizo patients | 10 | 2007 | 0.6 | Correa, MC^97^ | Journal of the American Academy of Dermatology | 12.8 | Mexico |
| 95 | Cutaneous hyperpigmentation induced by omeprazole mimicking ashy dermatosis | 11 | 2006 | 0.6 | Ramírez-Hernández, M^98^ | Journal of the European Academy of Dermatology and Venereology | 8.4 | Spain |
| 96 | Periorbital hyperpigmentation mimicking fixed drug eruption: a rare presentation of erythema dyschromicum perstans in a paediatric patient | 10 | 2006 | 0.6 | Sardana, K^99^ | Journal of the European Academy of Dermatology and Venereology | 8.4 | India |
| 97 | Linear hyperpigmentation with extensive epidermal apoptosis: A variant of linear lichen planus pigmentosus? | 11 | 2004 | 0.6 | Akagi, A^100^ | Journal of the American Academy of Dermatology | 12.8 | Japan |
| 98 | Erythema dyschromicum perstans: A case report and review | 13 | 2001 | 0.6 | Osswald, SS^101^ | Cutis | 2.1 | United States |
| 99 | Screening patch tests for pigmented contact dermatitis in Israel | 15 | 1999 | 0.6 | Trattner, A^102^ | Contact Dermatitis | 4.8 | Israel |
| 100 | Erythema dyschromicum perstans: response to dapsone therapy | 16 | 1998 | 0.6 | Kontochristopoulos, G^103^ | International Journal of Dermatology | 3.5 | Greece |

* The journal impact factor was collected from the Web of Science database.
